# Time series analysis and seasonality trends of SARS-CoV-2 in Ecuador (2020–2024): a four-year study

**DOI:** 10.3389/fpubh.2025.1621873

**Published:** 2025-12-17

**Authors:** Pablo Espinosa, Paulina Quirola-Amores, Saul Lema Asqui, Enrique Terán

**Affiliations:** 1 Universidad Internacional del Ecuador, Facultad de Ciencias de la Salud y la Vida, Escuela de Medicina, Grupo de Investigación Biomédico, Forense y Epidemiológico, Quito, Ecuador; 2 Escuela Salud Pública, Universidad San Francisco de Quito, Quito, Ecuador; 3 Instituto Microbiología, Universidad San Francisco de Quito, Quito, Ecuador; 4 Facultad de Medicina, Universidad de las Américas, Quito, Ecuador; 5 Colegio de Ciencias de la Salud, Universidad San Francisco de Quito USFQ, Quito, Ecuador

**Keywords:** SARS-CoV-2, COVID-19, seasonality, endemic, ARIMA, time series, case projection, Ecuador

## Abstract

**Introduction:**

Since the appearance of SARS-CoV-2 in 2019, the virus has been characterized by rapid spread and has generated multiple variants, creating ongoing challenges to healthcare systems worldwide. In Ecuador, reported COVID-19 cases declined steadily after 2022, falling from 10,677 cases in 2022 to 1,910 in 2023 and 720 in 2024. Reported deaths also decreased sharply, limited to 30 reported deaths in 2024, mainly reflecting the impact of vaccination programs. Although the WHO has declared that COVID-19 is no longer a global pandemic, it remains a public health concern requiring ongoing surveillance. Understanding whether SARS-CoV-2 is transitioning toward a seasonal endemic pattern remains complex, given its evolutionary dynamics, diverse clinical forms, and population-level factors. This study aimed to forecast seasonal trends and potential endemicity of SARS-CoV-2 in Ecuador using reported surveillance data from 2020 to 2024.

**Methods:**

A time series analysis was conducted using the endemic channel approach with ARIMA (p, d, q)(P, D, Q)[m] modeling, based on data from the Ecuadorian Ministry of Public Health.

**Results:**

The results showed an upward trend peaking in 2022, followed by stabilization in 2024. Consistent seasonal peaks occurred at the beginning of each year, followed by a gradual decline throughout the year. The ARIMA (0,2,1)(0,0,1)[52] model, validated through white noise tests, generated forecasts indicating a continued decline in case numbers.

**Discussion:**

These findings suggest that SARS-CoV-2 in Ecuador is adopting a secular, seasonal transmission pattern, potentially moderated by vaccination coverage.

## Introduction

SARS-CoV-2, a novel RNA virus from the Coronaviridae family, was first identified in Wuhan, China, in December 2019. Closely related to other pandemic-prone viruses such as SARS-CoV and MERS-CoV, it quickly spread worldwide. In March 2020, the World Health Organization (WHO) declared the COVID-19 pandemic a global health emergency. By the end of that year, over 183 million confirmed cases had been reported, underscoring its high transmissibility and substantial impact on public health systems (1, 2).

.Epidemiological and genomic analyses indicate that SARS-CoV-2 originated by zoonotic spillover, with subsequent mutations—particularly in the spike protein—leading to distinct viral variants. By late 2021, strains such as Alpha, Beta, Gamma, and Omicron had demonstrated increased transmissibility and partial immune escape, altered transmission dynamics, and prompted adjustments in surveillance and control strategies (3). Morbidity and mortality varied across regions, influenced by population density, healthcare capacity, and the timing of interventions. Urban areas experienced rapid early spread, with higher mortality in older adults and individuals with comorbidities during periods of limited treatment options and overwhelmed health systems, particularly during the initial phase when therapeutic options were limited, ICU capacity was strained, and clinical protocols were still evolving. As of the cutoff date for the study, 193.31 million confirmed COVID-19 cases and 3.04 million deaths had been reported globally. In Ecuador, between 2020 and 2024, 864,811 confirmed cases and 10,334 deaths were recorded (4–7). Over time, advances in clinical management reduced the severity and lethality of cases. However, excess mortality persisted, especially in low-and middle-income countries where vaccine access was limited, and health systems faced repeated surges (1, 3).

Vaccination played a pivotal role in controlling the COVID-19 pandemic. Accelerated research and emergency use authorizations enabled early access, significantly reducing SARS-CoV-2 transmission and mortality by 57% overall, particularly among high-risk populations. The BioNTech-Pfizer vaccine, the first deployed globally, demonstrated 95% efficacy, followed by AstraZeneca (90%) and Sinovac (79.3%) (8). The full impact became evident after the mass rollout: by the end of 2021, vaccination had prevented an estimated 19.8 million deaths and markedly reduced infections, hospitalizations, and severe cases (9).

Meta-analyses confirmed transmission reductions exceeding 40%, ICU admissions declining by more than 90%, and case fatality rates falling by nearly 70%. Therefore, global mortality from COVID-19 has steadily declined, from 4.3 million deaths in 2021 to 81,753 in 2024—a dramatic downward trend. In Ecuador, the 2021 vaccination campaign resulted in a greater than 40% reduction in cases; by 2022, peak infections had remained below 10,000, and mortality had become negligible. In countries with coverage of 30% or more, case numbers decreased by more than 40%. These outcomes highlight the effectiveness of sustained immunization in reducing disease burden and preventing future public health crises. In Ecuador, the first case of COVID-19 was reported in February 2020; within a few weeks, infections arose from 6–8 cases to over 60 as a consequence, national isolation measures were implemented as a control strategy, reaching 400 cases per month for 2020, 1,000 cases during 2021 and a mortality rate of 300 cases; showing the same pattern as worldwide showing a representative decreasing in it during 2022 and 2023 while in 2024 were 250 reported cases and only five deaths (5, 10–12).

Consequently, in March 2023, the WHO declared that COVID-19 was no longer a pandemic but a public health event of international concern. The organization urged countries worldwide to maintain surveillance and vaccination efforts to control outbreaks or potential epidemics of this disease, as it is not yet able to produce sustainable and long-lasting protection against SARS-CoV-2 infection; hence, the population remains susceptible to COVID-19. Furthermore, the seasonal behavior, the emergence of new SARS-CoV-2 variants, and the co-circulation with other respiratory viruses are also key considerations (13).

Five years into the pandemic, it is now possible to explore the potential seasonality or endemicity of SARS-CoV-2. However, classifying COVID-19 remains a complex and region-dependent process. According to the Centers for Disease Control and Prevention (CDC), such classification is challenging due to the emergence of new variants, fluctuating attack rates, and variable lethality. These factors contribute to an unpredictable transmission pattern, complicating reliable analysis and requiring robust data collection and sustained surveillance. Thus, SARS-CoV-2 has not yet demonstrated apparent seasonality and may continue to cause localized epidemics or even future pandemics (7, 14–16).

Understanding the seasonal dynamics of infectious diseases is essential for guiding vaccine strategies, including the optimal timing of booster dose deployment. It also addresses this public health priority and determines whether its trend would be adjusted in relation to other circulating respiratory viruses. The study aimed to analyze the seasonality of COVID-19 using time series methods, assess its potential transition to an endemic pattern, and develop a case projection model based on the ARIMA (p, d, q)(P, D, Q)[m] method in Ecuador from 2020 to 2024.

## Methods

### Data collection

This retrospective study analyzed COVID-19 case reports in Ecuador from 2020 to 2024, using official datasets from the Ministry of Public Health. The data included epidemiological week reports, confirmed and reported cases, regional distribution patterns, and mortality statistics (4–6). Data were systematically collected, formatted in XML and CSV, and archived using Microsoft Excel (v16.92). The study received ethical approval from the Ethics Committee of the International University of Ecuador (CEISH UIDE) under the code EX_2025_UIDE_PE (4–6).

### Endemic Channel analysis

A retrospective analytical methodology was employed to construct the endemic channel using collected SARS-CoV-2 data. Quartile-based stratification was applied to delineate four distinct zones: Q1: Secure Zone, Q2: Safety Zone, Q3: Alert Zone, and Q4: Epidemic Zone threshold. All confirmed COVID-19 cases were standardized and distributed across 52 epidemiological weeks and adjusted for population changes using Microsoft Excel (Version 16.92). Endemic channel graphs were subsequently generated using GraphPad Prism (Version 10.4.1) (17, 18).

### Statistical analysis and time series

A time series analysis was conducted to examine temporal trends in reported COVID-19 cases. Preliminary descriptive statistics were generated using GraphPad Prism (Version 10.4.1) to summarize data distribution. Data were organized into 52 epidemiological weeks per year and formatted into CSV for integration into RStudio (Version 2024.04.2 + 764). Following the methodology of Cuellar (19), the dataset was converted into a time series structure to evaluate seasonality, trends, and autoregressive components (19–22).

The Seasonal Autoregressive Integrated Moving Average model, expressed as ARIMA (p, d, q)(P, D, Q)[m], was employed to determine the optimal configuration and the necessary orders of differencing required for stationarity. The ndiffs() and nsdiffs() functions from the forecast package were applied to estimate non-seasonal (d) and seasonal (D) differencing, respectively. Model selection employed the auto.arima() function, using a stepwise search based on information criteria (AICc, BIC) and stationarity diagnostics (19–23).

In this framework, p, d, and q denote the autoregressive order, degree of differencing, and moving average order for the non-seasonal component. In contrast, P, D, and Q represent their seasonal counterparts. The parameter m was fixed at 52 to reflect annual seasonality in weekly data (19–23).

Model adequacy and residual behavior were evaluated using the Shapiro–Wilk test for normality, the Ljung–Box test for autocorrelation, and the Jarque–Bera test for skewness and kurtosis. If the data and residuals do not show normal parameters, a logarithmic transformation in base 10 is applied to normalize them. To assess forecast accuracy, the Diebold–Mariano test was used to test the null hypothesis of equal predictive accuracy across competing models. The final ARIMA (p, d, q)(P, D, Q)[m] model was then used to generate 52-week out-of-sample forecasts with 80 and 95% prediction intervals (19–23).

## Results

During the data collection period from 2020 to 2024, annual increases in reported SARS-CoV-2 cases were observed until 2022, after which infection rates declined. The Kruskal–Wallis test confirmed significant differences in case counts across years (*p* = 0.0001). The highest case burden occurred in 2022, when weekly reports exceeded 52,000. In contrast, 2024 showed markedly lower case levels, averaging 349.8 per week (90% CI: 240.1–459.6) (Table 1).

**Table 1:** Descriptive statistical analysis report of confirmed COVID-19 cases collected in Ecuador from 2020 to 2024.

Years	*n* (weeks)	Minimum confirmed cases	Maximum confirmed cases	Mean confirmed cases	Standard deviation	95% CI (min-max)		*p* (Kruskal–Wallis)
2020	52	1	8,883	4,077	2,662	3,336	4,818	0.0001
2021	52	1,561	14,234	7,381	4,278	6,189	8,572	
2022	52	341	52,300	8,338	11,824	5,046	11,630	
2023	52	148	2,749	638.2	443.1	514.8	761.6	
2024	31	14	1,130	349.8	299.2	240.1	459.6	

**Figure 1:**
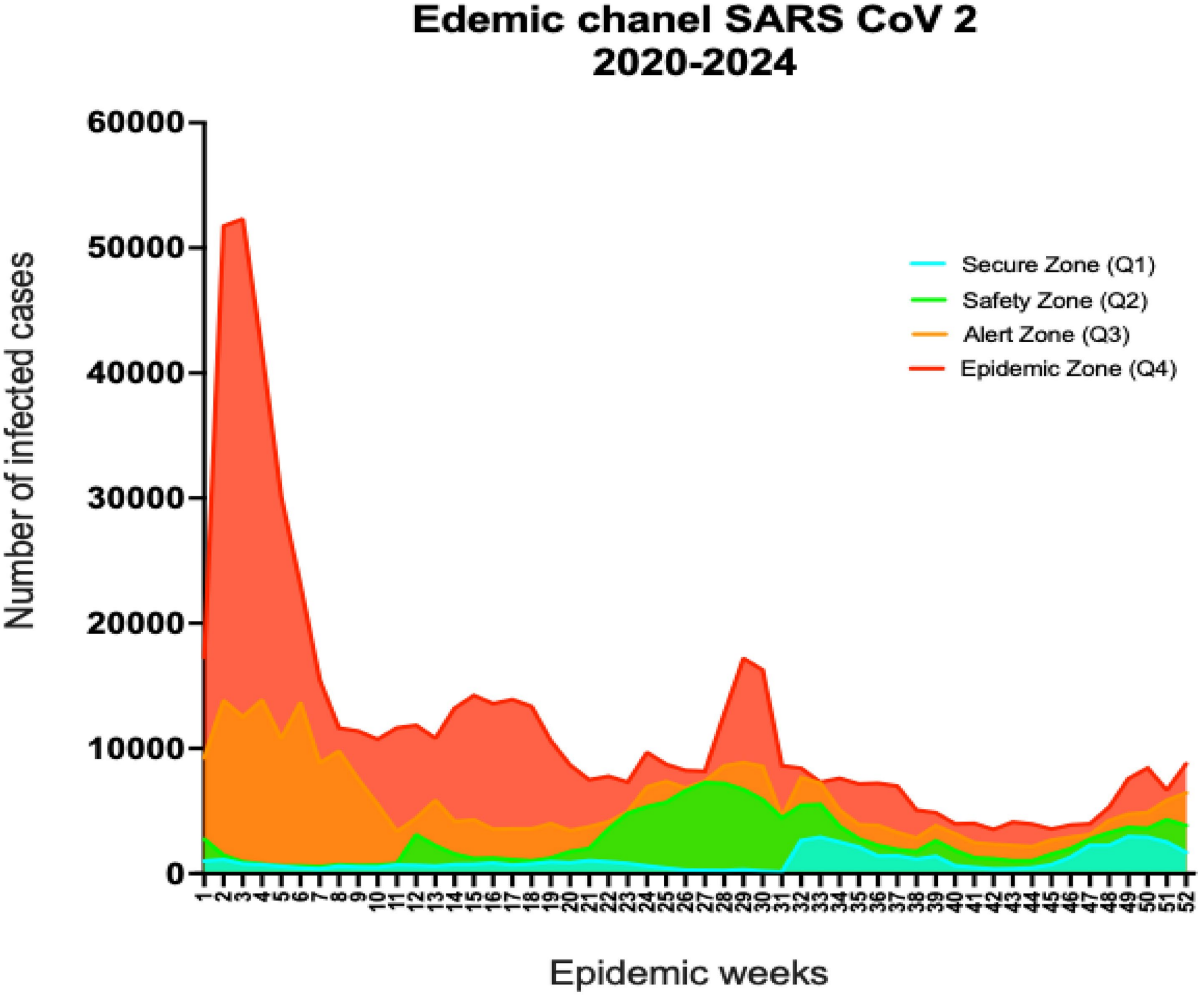
Endemic channel for SARS-CoV-2 in Ecuador. The endemic channel for SARS-CoV-2 in Ecuador covers 2020 to 2024 and is divided into epidemiological weeks. Quartile 1 (light blue) represents the “Secure Zone,” Quartile 2 (green) corresponds to the “Safety Zone,” Quartile 3 (orange) indicates the “Alert Zone,” and Quartile 4 (red) represents the “Epidemic Zone.”

Endemic channel analysis across the 5 years revealed consistent patterns. The highest incidence was concentrated in the early epidemiological weeks of each year, while the lowest case counts typically occurred between weeks 40 and 47, followed by a resurgence around week 49. Within the epidemic zone, weekly cases exceeded 50,000, whereas during weeks 28 to 30, they dropped to approximately 2,000. The success zone was narrow, with a maximum of only 450 cases between weeks 34 and 39 (Figure 1).

**Figure 2:**
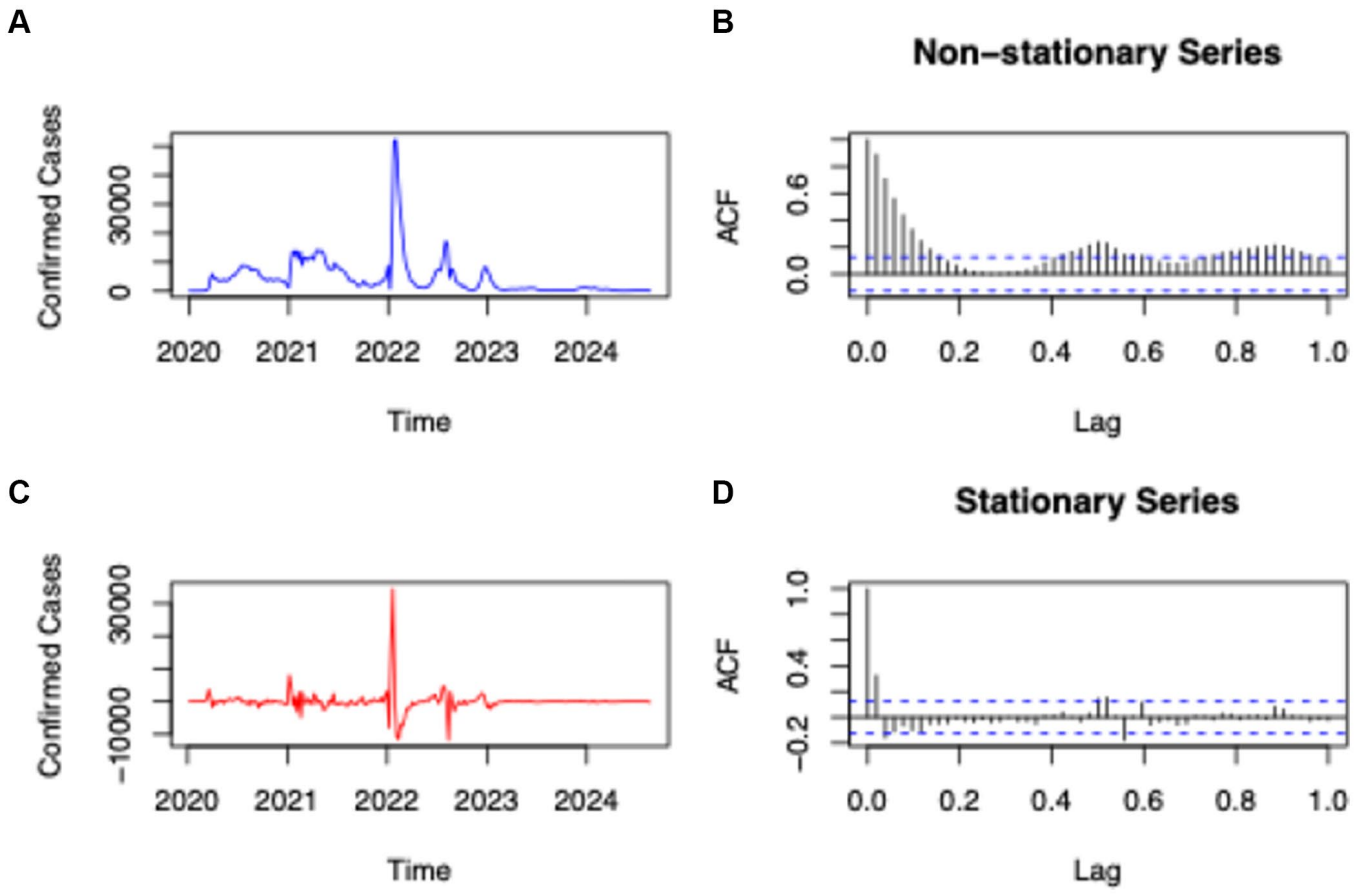
Time Series Analysis of SARS-CoV-2 Cases in Ecuador (2020–2024). **(a)** Presents the raw data of reported COVID-19 infections over time; **(b)** displays the autocorrelation model for the untransformed data; **(c)** depicts the transformation of raw COVID-19 infection data into a time series format; and **(d)** shows the autocorrelation model for the transformed data.

Seasonality analysis of the raw case data revealed seasonal patterns, with variance equal to zero. After transformation into a stationary time series, the mean and variance approached zero, indicating the presence of seasonality in the adjusted data. An exception occurred in 2022, when a marked increase in reported cases produced values outside the expected range. Autocorrelation analysis of the stationary series revealed a correlation among reported cases that decreased progressively with increasing lag, approaching the characteristics of white noise (Figure 2).

**Figure 3:**
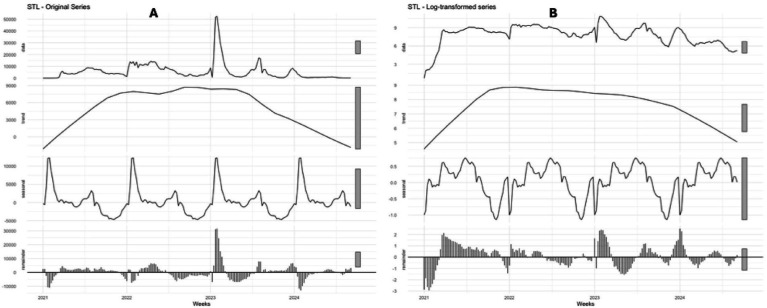
Decomposition, trend, and seasonality analysis of SARS-CoV-2 in Ecuador (2020–2024). **(a)** Shows the trend and seasonality analysis using the raw data of COVID-19 infections, and **(b)** displays the trend and seasonality analysis after transforming the COVID-19 infection data into a time series format using the log base 10 transformation in the data.

**Figure 4:**
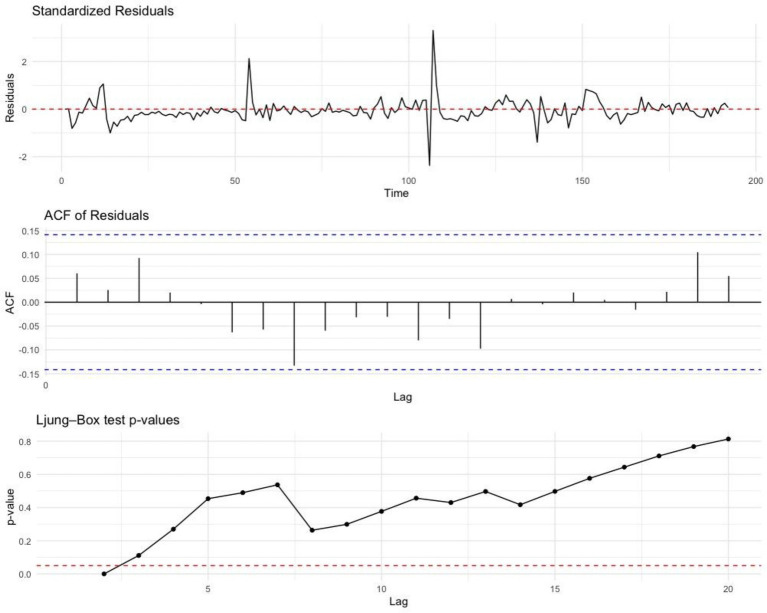
Residual plots for trend and seasonality analysis of SARS-CoV-2 in Ecuador (2020–2024). This figure presents the residual plots obtained from the trend and seasonality analysis of the SARS-CoV-2 time series data. The upper panel displays the time series data, the middle panel shows the autocorrelation plot, and the lower panel features the Ljung-Box plot.

**Figure 5:**
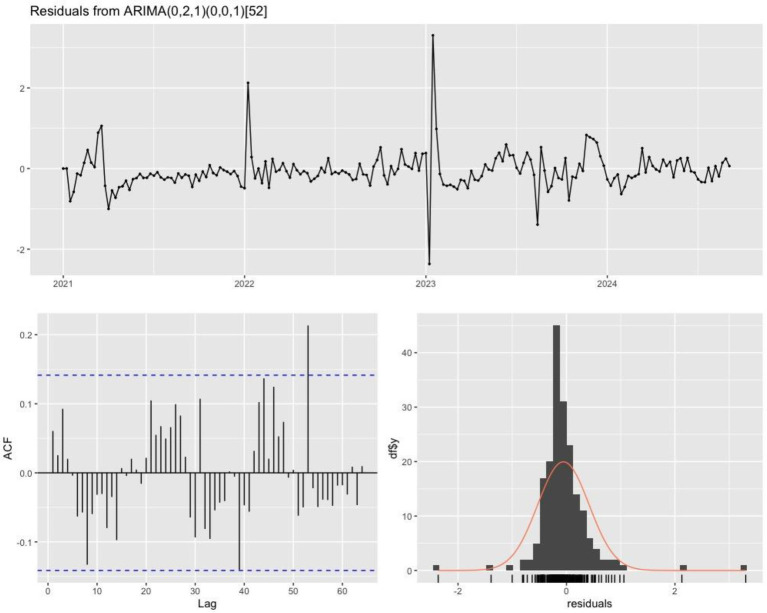
Moving average model seasonality for SARS-CoV-2 time series in Ecuador (2020–2024). It shows the analysis and ARIMA (p, d, q)(P, D, Q)[m] model generated for the transformed time series data of COVID-19 infections. The obtained model parameters were *p* = 0, d = 0, and q = 1, *p* = 0, D = 2, Q = 1, and m = 52, with an autoregressive component (AR) of 0.

**Figure 6:**
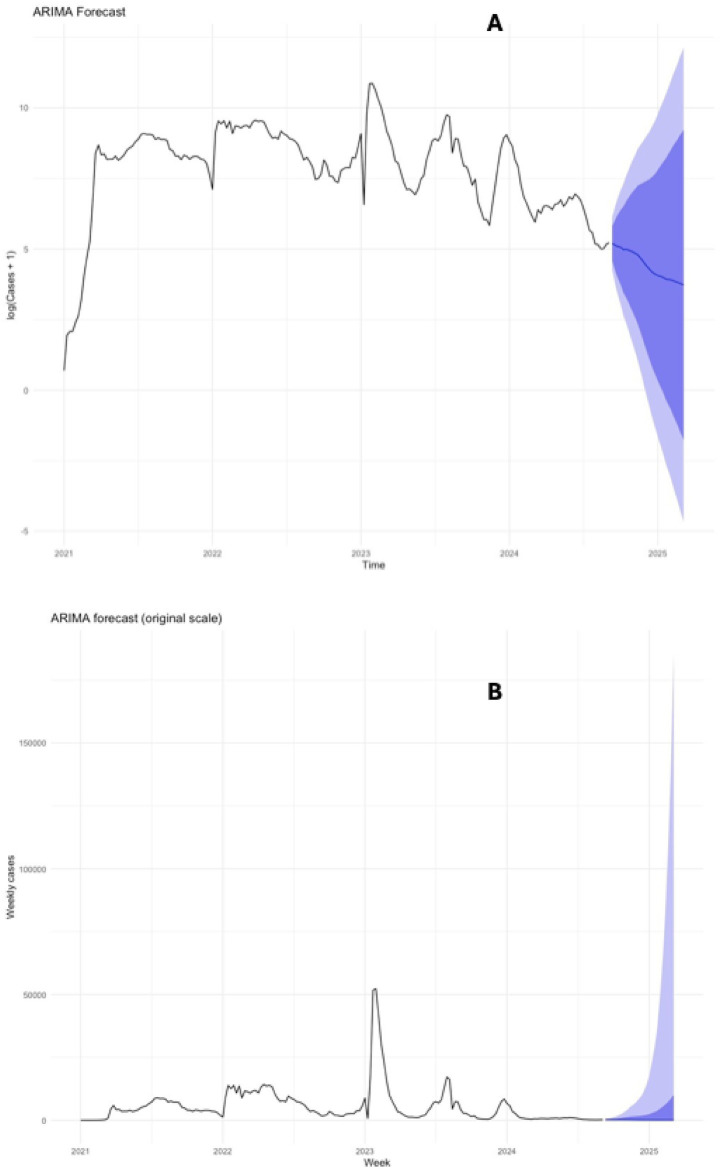
Forecast ARIMA (0,2,1)(0,0,1)[52] model: prediction Evaluation vs. Current SARS-CoV-2 Data in Ecuador (2020–2024), **(A)** Illustrates the predictive forecast model logarithmic estimating the potential number of cases for 2025 over the next 52 epidemiological weeks and **(B)** presents the projection graph for cases over the 52 weeks for 2025, compared with the original observed data.

The time series analysis of SARS-CoV-2 revealed a marked decline in infection rates beginning in 2023, stabilizing into a linear trend by late 2024. Since the onset of the pandemic in Ecuador, recurrent peaks have consistently occurred at the start of each year, particularly during epidemiological weeks 1 to 8, followed by a smaller peak between weeks 20 and 28 and a gradual decline toward the end of the year. This secular seasonal pattern remained stable across the study period (Figure 3; Table 2). Model comparisons indicated that ETS produced lower error values than ARIMA (0,2,1)(0,0,1)[52], while STL + ETS further reduced errors but exhibited unusually high MAPE values. The ARIMA (0,2,1)(0,0,1)[52] model achieved in-sample performance comparable to ETS, but external validation revealed substantially higher error metrics (Tables 3, 4; Figures 4–6).

**Table 2:** Residual analysis and moving average statistics of the ARIMA (0,2,1) (0,0,1)[52] model for SARS-CoV-2 in Ecuador (2020–2024).

Variables	Estimated	Standard Error	*p*	Sigma^2^	Likelihood	AIC	AICc	BIC	
Ma1	−0.9449	0.0380		0.0000					
Sma1	−0.0901	0.0869		0.2998					
ARIMA				0.2307	−130.59		267.17	267.3	276.91

AR refers to the autocorrelation coefficient, MA denotes the moving average, AIC is the Akaike Information Criterion, AICc is the corrected version for small sample sizes, BIC represents the Bayesian Information Criterion, and Ndiffs indicates the number of regular (non-seasonal) differences required to achieve stationarity in a time series.

**Table 3:** Normality tests applied to the original data and to the residuals of the ARIMA (0,2,1) (0,0,1)[52] model after log10 transformation.

Test	Original data	Residuals
Shapiro Wilk (*p*)	0.00002	0.00000013
Jarque Bera (*p*)	0.00002	0.000000022
Kolmogorov–Smirnov (*p*)		0.00242
Anderson-Darling (*p*)		0.000000016

**Table 4:** Performance of seasonality models.

Models	MAE	RMSE	MAPE
ETS	0.275	0.504	4.67%
STL + ETS	0.275	0.482	4.33%

MAE, Mean Absolute Error; RMSE, Root Mean Square Error; MAPE, Mean Absolute Percentage Error, ETS Error; Trend, Seasonality (Modelo de Holt–Winters/ETS), STL Seasonal-Trend decomposition using Loess.

Residual diagnostics revealed significant deviations from normality, both in the original dataset and in the residuals of the fitted model, even after applying a base-10 logarithmic transformation. This was confirmed by multiple normality tests, including the Shapiro–Wilk, Kolmogorov–Smirnov, Anderson–Darling, and Jarque–Bera tests, all of which yielded *p*-values < 0.001 (Table 3). Despite this, the Ljung–Box tests for residual autocorrelation were non-significant (*p* = 0.4611), as shown in Table 5, indicating that the residuals behaved like white noise and suggesting the model adequately captured the temporal structure of the series.

**Table 5:** Independence tests applied to residuals of the ARIMA (0,2,1) (0,0,1)[52] model for weekly SARS-CoV-2 cases in Ecuador, 2020–2024.

Test	Q	df	*p*	Model df	Total lag
Box-Ljung	8.6019	8	0.3770	2	10
Ljung-Box	32.1133	32	0.4611	2	34

**Figure 7:**
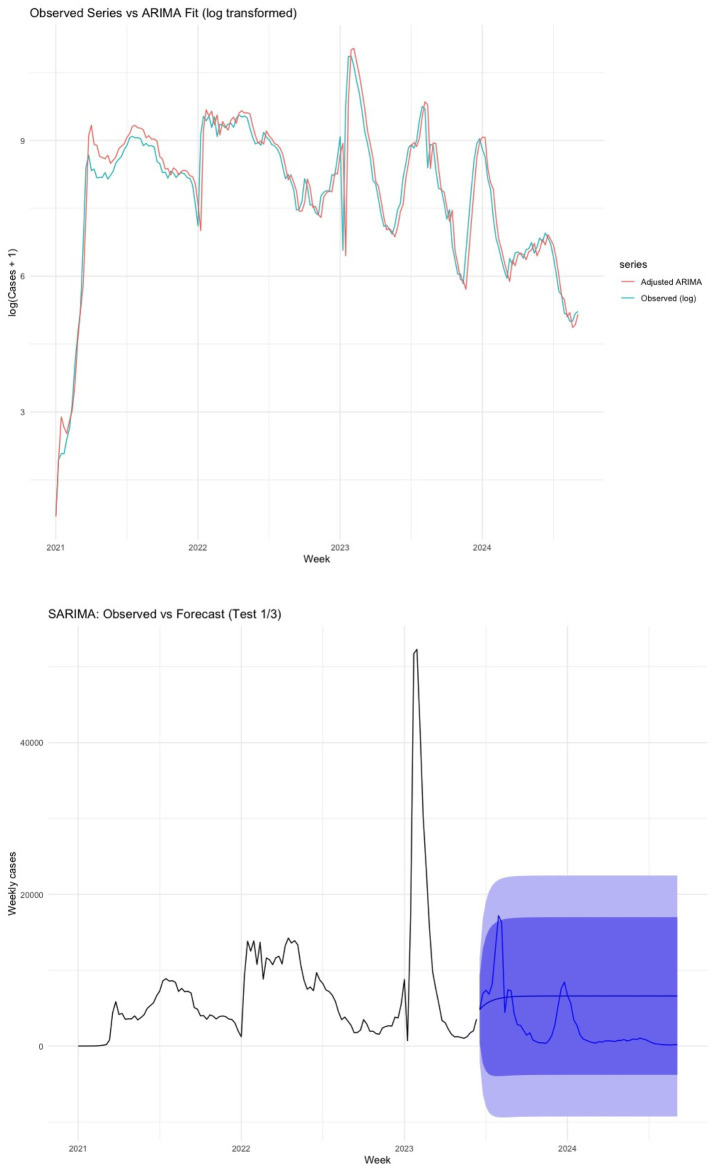
Forecast and seasonality from the ARIMA (0,2,1)(0,0,1)[52] model. **(A)** Observed weekly COVID-19 cases (light blue) and fitted values with forecasts (red). **(B)** Projected cases over a 52-week horizon based on the ARIMA model (blue) compared with observed data.

The fitted ARIMA (0,2,1)(0,0,1)[52] model successfully replicated the general pattern of the epidemic curve in the training set, capturing moderate fluctuations while underestimating extreme peaks. Forecast outputs preserved the observed cyclical dynamics with broad 80 and 95% confidence intervals. However, out-of-sample validation results (Table 6; Figure 7) revealed higher error metrics, reflecting the challenges in predicting periods of sudden epidemiological change (Table 7).

**Table 6:** Out-of-sample validation analysis.

Model	MAE	RMSE	MAPE	sMAPE	MASE
ARIMA test	1.489	1.730	23.53%	20.21%	0.895

MAE, Mean Absolute Error; RMSE, Root Mean Square Error; MAPE, Mean Absolute Percentage Error; sMAPE, Symmetric Mean Absolute Percentage Error; MASE, Mean Absolute Scaled Error.

**Table 7:** Diebold-Mariano test comparing ETS and STL + ETS.

Horizont (h)	DM (*p*) MAE	DM (p) RMSE
H = 1	0.2315	0.8184
H = 6	0.4541	0.0693
H = 12	0.0314	0.0038

MAE, Mean Absolute Error, RMSE Root Mean Square Error, H the time of forecasts in weeks.

## Discussion

By the end of 2024, more than 777 million COVID-19 cases had been reported worldwide, with a notable decline in incidence following global peaks in late 2022. Ecuador mirrored this trajectory, reporting its highest national incidence in 2022 with 52,300 cases, followed by a steady reduction through 2024. These national patterns are consistent with WHO reports, which document global declines during the same period (3, 24–29).

Time series analysis of Ecuadorian data between 2020 and 2024 revealed consistent cyclical patterns, characterized by dual seasonal peaks. The ARIMA (0,2,1)(0,0,1)[52] model effectively captured these dynamics, detecting a primary surge from January to March and a secondary rise from May to July. Endemic channel analysis reinforced these findings, demonstrating that transmission is becoming more regular and predictable. The persistence of this dual-peak structure over five consecutive years suggests that SARS-CoV-2 is undergoing seasonal adaptation, likely influenced by the emergence of new variants, increasing vaccine coverage, and progressive development of population-level immunity (27, 30–32).

According to the epidemiology of transmissible diseases, the temporal series would represent seasonal variations as regular fluctuations each year, secular patterns with multiple peaks during the year, or secular trends that persist over a long period of time. In our study of SARS-CoV-2 in Ecuador (2020–2024), even though the ARIMA (0,2,1)(0,0,1)[52] led to assuming a weekly seasonality of 52 weeks per year, a practice nowadays accepted for practical epidemiological analysis, a secular pattern was highlighted by a progressive and maintained decrease in the reported COVID-19 cases during 2021. This mentioned behavior would be explained by the massive vaccination campaigns, the progressive acquired immunity (natural or vaccine-induced), and the appearance of other SARS-CoV-2 strains that showed less virulence and adaptation, transforming the dynamic of transmission through the country. Therefore, the established model effectively captures the secular trend with transient epidemic cycles, in contrast to a strong and marked annual pattern (33–36).

These seasonal dynamics parallel the behavior of influenza viruses, which typically peak in winter in temperate regions and display biannual peaks in tropical and subtropical settings. Influenza A (H3N2) usually peaks earlier in the season, while influenza B often drives secondary waves later in the year (37–39). Although SARS-CoV-2 lacks the rapid antigenic drift characteristic of influenza, its increasingly regular transmission cycles suggest meaningful epidemiological parallels. Recognizing these similarities can strengthen surveillance and guide seasonally timed vaccination strategies (40–44).

Comparison with SARS-CoV-1 underscores differences in long-term dynamics. While both viruses caused sharp surges after their emergence, SARS-CoV-1 outbreaks were contained by 2005 and remained geographically limited. In contrast, SARS-CoV-2 has continued to circulate globally. Its higher transmissibility, supported by spike protein mutations that enhance ACE2 receptor binding and facilitate immune evasion, together with factors such as international connectivity and urban density, contributed to its broader and more persistent spread (24, 26, 45–47).

Endemic channel analysis confirmed that in Ecuador, case counts exceeded the endemic threshold during the first epidemiological weeks of each year, followed by declines into the endemic zone by mid-year. Yet, the subnational assessment was limited by data constraints. Heterogeneity in healthcare access, intervention timing, and population density across provinces may influence local transmission patterns and should be considered in future analyses (27, 29, 48–50). Vaccination campaigns launched in 2021 substantially reduced transmission, but high case numbers persisted into 2022, reflecting delays in vaccine coverage and the emergence of novel variants. The decline observed from 2023 onward highlights the impact of population-level immunity but also suggests that Ecuador transitioned more slowly than other settings (9, 13, 51, 52).

Forecasting results provided additional insights. ARIMA models were accurate in the short term, predicting early 2025 peaks of 240 to 250 cases per week, followed by rapid declines to near-zero levels. However, forecasts beyond 1 year consistently projected zero incidence, underscoring ARIMA’s reduced sensitivity to long-range uncertainty and inability to capture the stochastic nature of epidemics. Similar findings have been reported in Brazil and Romania, where ARIMA models performed well over short horizons but failed at longer ones (16, 31, 53–55). ARIMA validation confirmed stronger long-term performance. Diagnostic tests showed independent residuals consistent with white noise, confirming model adequacy despite the absence of normally distributed errors, which is common in epidemiological series (56–59).

Residual diagnostic tests revealed that the fitted ARIMA (0,2,1)(0,0,1)[52] model did not fully meet the assumption of normality, even after applying a logarithmic transformation to the original series. All normality tests returned statistically significant results (*p* < 0.001), indicating deviations from a normal distribution in the residuals. However, such deviations are often observed in epidemiological time series of infectious diseases, where residual patterns may be influenced by factors such as reporting lags, outbreak clusters, or sudden epidemic peaks. In contrast, the Ljung-Box test yielded non-significant results (*p* = 0.377 and *p* = 0.461), suggesting no autocorrelation and supporting the assumption of independence, which aligns with white noise. The model effectively captured the overall downward trend of SARS-CoV-2 cases and represented the major epidemic waves, although it tended to underestimate extreme peaks.

Out-of-sample validation, using one-third of the data, showed moderate increases in forecast error metrics (MAE, RMSE, MAPE), which is expected given the heightened variability during post-peak epidemic phases. Despite the observed deviations from normality, the independence of the residuals and the model’s ability to replicate seasonal and secular trends support its relevance and validity in epidemiological applications (56–59).

Limitations remain, as case reporting in Ecuador has been affected by variability in testing capacity, evolving diagnostic criteria, and incomplete records, particularly in the early pandemic years. These issues limit the reliability of complex forecasting models. Machine learning approaches, such as LSTM and recurrent neural networks, can capture nonlinearities; however, their effectiveness is constrained by small, noisy datasets and a lack of transparency (27, 30–32, 60). By contrast, ARIMA-based approaches offer greater interpretability, allowing for decomposition into trend and seasonal components, which is essential for epidemiological applications in data-limited contexts (61–63).

Despite reduced incidence across Latin America, SARS-CoV-2 continues to pose challenges due to evolving variants and seasonal surges. The recurrent dual-peak pattern observed in Ecuador supports a gradual shift toward endemicity, though the process remains incomplete. Continued transmission variability highlights the need for caution in defining SARS-CoV-2 as entirely endemic (24, 26, 45–47).

Between 2020 and 2024, SARS-CoV-2 transmission in Ecuador exhibited increasingly regular seasonal peaks, suggesting a progressive transition toward endemicity. Nevertheless, uncertainties related to data quality, viral evolution, and regional disparities require cautious interpretation. Vaccination significantly reduced incidence but is insufficient alone to stabilize transmission. Enhanced surveillance during high-risk seasonal periods is necessary to prevent resurgence. Future research should integrate high-resolution, real-time data and explore hybrid models that combine the transparency of traditional time series with the adaptability of explainable machine learning. Continued monitoring and multidisciplinary approaches will be essential to anticipate transmission dynamics and guide effective public health responses.

## Data Availability

The raw data supporting the conclusions of this article will be made available by the authors, without undue reservation.

## References

[1] Chatterjee S Bhattacharya M Nag S Dhama K Chakraborty C. A detailed overview of SARS-CoV-2 omicron: its sub-variants, mutations and pathophysiology, clinical characteristics, immunological landscape, immune escape, and therapies. Viruses. (2023) 15:1–27. doi: 10.3390/v15010167 PMC9866114 36680207

[2] Santos-López G Cortés-Hernández P Vallejo-Ruiz V Reyes-Leyva J. Sars-cov-2: basic concepts, origin and treatment advances. Gac Med Mex. (2021) 157:84–9. doi: 10.24875/GMM.M21000524, 34125824

[3] Thirumugam G. Radhakrishnan Y. Ramamurthi S. (2020). Since January 2020 Elsevier has created a COVID-19 resource Centre with free information in English and mandarin on the novel coronavirus COVID-19. The COVID-19 resource Centre is hosted on Elsevier connect, the company's public news and information. January.

[4] MSP (2021). Datos epidemiológicos COVID 19. MSP Available online at: https://app.powerbi.com/view?r=eyJrIjoiY2ExMGM3NTAtM2Q5MC00ZjRkLTk2NzUtNmFkM2Q3NGIxZTEwIiwidCI6ImQxMDMxZjJkLWI0MzAtNDMwOS04ZGFhLThhMDdmYzJiODE2ZCIsImMiOjR9 (Accessed April 30, 2025).

[5] OSE (2021). Ecuador | monitoreo de casos de pandemia covid-19 (coronavirus). Observatorio Social Del Ecuador. Available online at: https://www.covid19ecuador.org/ecuador (Accessed April 30, 2025).

[6] PAHO (2021). SARS CoV2 Situation - Region of the Americas - PAHO/WHO | Pan American Health Organization. Available online at: https://www.paho.org/en/covid-19-weekly-updates-region-americas (Accessed April 30, 2025).

[7] Parums DV. Editorial: factors driving new variants of SARS-CoV-2, immune escape, and resistance to antiviral treatmentsas the end of the COVID-19 pandemic is declared. Med Sci Monit. (2023) 29:4–6. doi: 10.12659/MSM.942960, 37908161 PMC10626992

[8] Kyriakidis NC López-Cortés A González EV Grimaldos AB Prado EO. SARS-CoV-2 vaccines strategies: a comprehensive review of phase 3 candidates. Npj Vaccin. (2021) 6:28. doi: 10.1038/s41541-021-00292-w, 33619260 PMC7900244

[9] Watson OJ Barnsley G Toor J Hogan AB Winskill P Ghani AC. Global impact of the first year of COVID-19 vaccination: a mathematical modelling study. Lancet Infect Dis. (2022) 22:1293–302. doi: 10.1016/S1473-3099(22)00320-6, 35753318 PMC9225255

[10] COE (2021). Resoluciones COE Nacional 21 de abril de 2021 – Servicio Nacional de Gestión de Riesgos y Emergencias. COE. Available online at: https://www.gestionderiesgos.gob.ec/resoluciones-coe-nacional-21-de-abril-de-2021/ (Accessed April 30, 2025).

[11] MSP (2020). SITUACIÓN NACIONAL por COVID-19 (coronavirus). 1

[12] Nguyen NN Nguyen YN Hoang VT Million M Gautret P. SARS-CoV-2 reinfection and severity of the disease: a systematic review and meta-analysis. Viruses. (2023) 15:1–11. doi: 10.3390/v15040967 PMC10145185 37112949

[13] Singh P Anand A Rana S Kumar A Goel P Kumar S . Impact of COVID-19 vaccination: a global perspective. Front Public Health. (2023) 11:1–10. doi: 10.3389/fpubh.2023.1272961 PMC10808156 38274537

[14] CDC. (2013). SARS Response Timeline. CDC. Available online at: https://archive.cdc.gov/www_cdc_gov/about/history/sars/timeline.htm (Accessed April 30, 2025).

[15] Prasad V Haslam A. COVID-19 vaccines: history of the pandemic's great scientific success and flawed policy implementation. Monash Bioeth Rev. (2024) 42:28–54. doi: 10.1007/s40592-024-00189-z, 38459404 PMC11368972

[16] Sarker R Roknuzzaman ASM Nazmunnahar Shahriar M Hossain MJ Islam MR. The WHO has declared the end of pandemic phase of COVID-19: way to come back in the normal life. Health Sci Rep. (2023) 6:1–5. doi: 10.1002/hsr2.1544 PMC10478644 37674622

[17] Aguilar MS Castaneda A. What mathematical competencies does a citizen needs to interpret Mexico's official information about the COVID-19 pandemic? Educ Stud Math. (2021) 108:227–48. doi: 10.1007/s10649-021-10082-9, 34934238 PMC8299177

[18] Jaramillo C Martínez J In: Moderno M, editor. Epidemiologia Veterinaria. Mexico (Mexico): Manueal Moderno (2010).

[19] Cuellar L. (2020). Script estacionarias serie. Tecnológico de Monterrey. Available online at: https://www.mediafire.com/file/yb9otg1y66v0vxy/script+estacionarias.docx/file (Accessed April 30, 2025).

[20] Raftery AE. Time series analysis. Eur J Oper Res. (1985) 20:127–37. doi: 10.1016/0377-2217(85)90052-9 (Accessed April 30, 2025).

[21] RPubs. (2017). RPubs - Series Temporales. Rstudio. Available online at: https://rpubs.com/palominoM/series (Accessed April 30, 2025).

[22] RPubs (2021). RPubs - Series de Tiempo. Rstudio Available online at: https://rpubs.com/revite19/749499 (Accessed April 30, 2025).

[23] Bajaj A. (2024). ARIMA & SARIMA: real-world time series forecasting. NeptuneAi. Available online at: https://neptune.ai/blog/arima-sarima-real-world-time-series-forecasting-guide (Accessed April 30, 2025).

[24] Kirtipal N Bharadwaj S Kang SG. From SARS to SARS-CoV-2, insights on structure, pathogenicity and immunity aspects of pandemic human coronaviruses. Infect Genet Evol. (2020) 85:104502. doi: 10.1016/j.meegid.2020.104502, 32798769 PMC7425554

[25] Markov PV Ghafari M Beer M Lythgoe K Simmonds P Stilianakis NI . The evolution of SARS-CoV-2. Nat Rev Microbiol. (2023) 21:361–79. doi: 10.1038/s41579-023-00878-2, 37020110

[26] Pekar JE Magee A Parker E Moshiri N Izhikevich K Havens JL . The molecular epidemiology of multiple zoonotic origins of SARS-CoV-2. Science. (2022) 377:960–6. doi: 10.1126/science.abp8337, 35881005 PMC9348752

[27] Somyanonthanakul R Warin K Amasiri W Mairiang K Mingmalairak C Panichkitkosolkul W . Forecasting COVID-19 cases using time series modeling and association rule mining. BMC Med Res Methodol. (2022) 22:281–18. doi: 10.1186/s12874-022-01755-x, 36316659 PMC9624022

[28] Woo TM. 2009 H1N1 influenza pandemic. J Pediatr Health Care. (2010) 24:258–66. doi: 10.1016/j.pedhc.2010.05.001, 20620852

[29] World Health Organisation (2022). Global report on infection prevention and control.

[30] Chung H-Y Jian M-J Chang C-K Lin J-C Yeh K-M Chen C-W . Novel dual multiplex real-time RT-PCR assays for the rapid detection of SARS-CoV-2, influenza a/B, and respiratory syncytial virus using the BD MAX open system. Emerg Microb Infect. (2021) 10:161–6. doi: 10.1080/22221751.2021.1873073, 33410371 PMC7832498

[31] Huergo LF Paula NM Gonçalves ACA Kluge CHS Marins PHSA Camargo HSC . SARS-CoV-2 seroconversion in response to infection and vaccination: a time series local study in Brazil. Microbiol Spectr. (2022) 10:1–7. doi: 10.1128/spectrum.01026-22 PMC9430992 35770982

[32] Kumar N. Susan S. (2020).” COVID-19 pandemic prediction using time series forecasting models.” in 2020 11th International Conference on Computing, Communication and Networking Technologies, ICCCNT 2020.

[33] Baek K Park C. Analyzing the dynamics of complicated and uncomplicated appendicitis during the COVID-19 pandemic in Seoul, Korea: a multifaceted time series approach. Epidemiol Health. (2024) 46:e2024081. doi: 10.4178/epih.e2024081, 39363604 PMC11832239

[34] Brachman PS In: Baron S, editor. Medical Microbiology, vol. 12. 4th ed. Galveston (TX): University of Texas Medical Branch at Galveston (1996). 21413252

[35] Gaviria A Tamayo-Trujillo R Paz-Cruz E Cadena-Ullauri S Guevara-Ramírez P Ruiz-Pozo VA . Assessment of the COVID-19 pandemic progression in Ecuador through seroprevalence analysis of anti-SARS-CoV-2 IgG/IgM antibodies in blood donors. Front Cell Infect Microbiol. (2024) 14:1373450. doi: 10.3389/fcimb.2024.1373450, 38975325 PMC11224293

[36] Mahrokhian SH Tostanoski LH Vidal SJ Barouch DH. COVID-19 vaccines: immune correlates and clinical outcomes. Hum Vaccin Immunother. (2024) 20:4549. doi: 10.1080/21645515.2024.2324549, 38517241 PMC10962618

[37] CDC. (2024). Flu season. CDC.

[38] Gavigan P McCullers JA. Influenza: annual seasonal severity. Curr Opin Pediatr. (2019) 31:112–8. doi: 10.1097/MOP.0000000000000712, 30480557

[39] WHO (2025). Influenza (seasonal) WHO.

[40] Albicócco AP Vezzani D. Further study on Ascogregarina culicis in temperate Argentina: prevalence and intensity in *Aedes aegypti* larvae and pupae. J Invertebr Pathol. (2009) 101:210–4. doi: 10.1016/j.jip.2009.05.003, 19450603

[41] Aldeyab MA Monnet DI López-Lozano JM Hughes CM Scott MG Kearney MP . Modelling the impact of antibiotic use and infection control practices on the incidence of hospital-acquired methicillin-resistant *Staphylococcus aureus*: a time-series analysis. J Antimicrob Chemother. (2008) 62:593–600. doi: 10.1093/jac/dkn198, 18467307

[42] Keilman LJ. Seasonal influenza (flu). Nurs Clin North Am. (2019) 54:227–43. doi: 10.1016/j.cnur.2019.02.009, 31027663

[43] Park JE Ryu Y. Transmissibility and severity of influenza virus by subtype. Infect Genet Evol. (2018) 65:288–92. doi: 10.1016/j.meegid.2018.08.007, 30103034

[44] Tamerius J Nelson MI Zhou SZ Viboud C Miller MA Alonso WJ. Global influenza seasonality: reconciling patterns across temperate and tropical regions. Environ Health Perspect. (2011) 119:439–45. doi: 10.1289/ehp.1002383, 21097384 PMC3080923

[45] Chakraborty H Bhattacharjya S. Mechanistic insights of host cell fusion of SARS-CoV-1 and SARS-CoV-2 from atomic resolution structure and membrane dynamics. Biophys Chem. (2020) 265:106438. doi: 10.1016/j.bpc.2020.106438, 32721790 PMC7375304

[46] Primorac D Vrdoljak K Brlek P Pavelić E Molnar V Matišić V . Adaptive immune responses and immunity to SARS-CoV-2. Front Immunol. (2022) 13:1–13. doi: 10.3389/fimmu.2022.848582 PMC9114812 35603211

[47] Sarkar JP Saha I Seal A Maity D Maulik U. Topological analysis for sequence variability: case study on more than 2K SARS-CoV-2 sequences of COVID-19 infected 54 countries in comparison with SARS-CoV-1 and MERS-CoV. Infect Genet Evol. (2021) 88:104708. doi: 10.1016/j.meegid.2021.104708, 33421654 PMC7787073

[48] Leiva V Alcudia E Montano J Castro C. An epidemiological analysis for assessing and evaluating COVID-19 based on data analytics in Latin American countries. Biology. (2023) 12:1–21. doi: 10.3390/biology12060887 PMC10295742 37372171

[49] Londoño-Ruiz JP Gutierrez-Tobar IF Bermúdez-Bohórquez NL Rodríguez AE. First publication of endemic channels as part of a pediatric antimicrobial stewardship program: when to turn on the alarms? Recommendations of a pediatric ASP program. BMC Infect Dis. (2023) 23:21–8. doi: 10.1186/s12879-022-07916-z, 36631755 PMC9833633

[50] Messacar K Baker RE Park SW Nguyen-Tran H Cataldi JR Grenfell B. Preparing for uncertainty: endemic paediatric viral illnesses after COVID-19 pandemic disruption. Lancet. (2022) 400:1663–5. doi: 10.1016/S0140-6736(22)01277-6, 35843260 PMC9282759

[51] Shrestha NK Burke PC Nowacki AS Gordon SM. Effectiveness of the 2023–2024 formulation of the COVID-19 messenger RNA vaccine. Clin Infect Dis. (2024) 79:405–11. doi: 10.1093/cid/ciae132, 38465901

[52] Ulrichs T Rolland M Wu J Nunes MC El Guerche-Séblain C Chit A. Changing epidemiology of COVID-19: potential future impact on vaccines and vaccination strategies. Expert Rev Vaccines. (2024) 23:510–22. doi: 10.1080/14760584.2024.2346589, 38656834

[53] Periwal N Rathod SB Sarma S Johar GS Jain A Barnwal RP . Time series analysis of SARS-CoV-2 genomes and correlations among highly prevalent mutations. Microbiol Spect. (2022) 10:e0121922–1. doi: 10.1128/spectrum.01219-22, 36069583 PMC9603882

[54] Thomas S Machuel P Foubert J Nafilyan V Bannister N Colvin H . Study protocol for the use of time series forecasting and risk analyses to investigate the effect of the COVID-19 pandemic on hospital admissions associated with new-onset disability and frailty in a national, linked electronic health data setting. BMJ Open. (2023) 13:e067786. doi: 10.1136/bmjopen-2022-067786, 37208137 PMC10201261

[55] Wang P Zheng X Ai G Liu D Zhu B. Time series prediction for the epidemic trends of COVID-19 using the improved LSTM deep learning method: case studies in Russia, Peru and Iran. Chaos, Solitons Fractals. (2020) 140:110214. doi: 10.1016/j.chaos.2020.110214, 32839643 PMC7437443

[56] Li Y Li X Lan X Xue C Zhang B Wang YB. Impact of COVID-19 on epidemic trend of hepatitis C in Henan Province assessed by interrupted time series analysis. BMC Infect Dis. (2023) 23:691. doi: 10.1186/s12879-023-08635-9, 37848842 PMC10580576

[57] Man H Huang H Qin Z Li Z. Analysis of a SARIMA-XGBoost model for hand, foot, and mouth disease in Xinjiang, China. Epidemiol Infect. (2023) 151:e200. doi: 10.1017/S0950268823001905, 38044833 PMC10729004

[58] Perez-Guerra UH Macedo R Manrique YP Condori EA Gonzáles HI Fernández E . Seasonal autoregressive integrated moving average (SARIMA) time-series model for milk production forecasting in pasture-based dairy cows in the Andean highlands. PLoS One. (2023) 18:e288849. doi: 10.1371/journal.pone.0288849, 37972120 PMC10653396

[59] Wang Y Xu C Zhang S Wang Z Yang L Zhu Y . Temporal trends analysis of tuberculosis morbidity in mainland China from 1997 to 2025 using a new SARIMA-NARNNX hybrid model. BMJ Open. (2019) 9:e024409. doi: 10.1136/bmjopen-2018-024409, 31371283 PMC6678063

[60] Zrieq R Kamel S Boubaker S Algahtani FD Alzain MA Alshammari F . Time-series analysis and healthcare implications of COVID-19 pandemic in Saudi Arabia. Healthcare (Switzerland). (2022) 10:1–27. doi: 10.3390/healthcare10101874 PMC9601417 36292321

[61] Fernández-Naranjo RP Vásconez-González E Simbaña-Rivera K Gómez-Barreno L Izquierdo-Condoy JS Cevallos-Robalino D . Statistical data driven approach of COVID-19 in Ecuador: R0 and Rt estimation via new method. Infect Dis Model. (2021) 6:232–43. doi: 10.1016/j.idm.2020.12.012, 33506154 PMC7811040

[62] Gutierrez L. de Medrano R. Aznarte J. L. (2021). COVID-19 forecasting with deep learning: a distressing survey. 0–18.

[63] Moein S Nickaeen N Roointan A Borhani N Heidary Z Javanmard SH . Inefficiency of SIR models in forecasting COVID-19 epidemic: a case study of Isfahan. Sci Rep. (2021) 11:4725–9. doi: 10.1038/s41598-021-84055-6, 33633275 PMC7907339

